# Phylogenomics with incomplete taxon coverage: the limits to inference

**DOI:** 10.1186/1471-2148-10-155

**Published:** 2010-05-25

**Authors:** Michael J Sanderson, Michelle M McMahon, Mike Steel

**Affiliations:** 1 Department of Ecology and Evolutionary Biology, University of Arizona, Tucson AZ 85721 USA; 2 Department of Plant Sciences, University of Arizona, Tucson AZ 85721 USA; 3 Biomathematics Research Centre, University of Canterbury, Christchurch, New Zealand

## Abstract

**Background:**

Phylogenomic studies based on multi-locus sequence data sets are usually characterized by partial taxon coverage, in which sequences for some loci are missing for some taxa. The impact of missing data has been widely studied in phylogenetics, but it has proven difficult to distinguish effects due to error in tree reconstruction from effects due to missing data per se. We approach this problem using a explicitly phylogenomic criterion of success, *decisiveness*, which refers to whether the pattern of taxon coverage allows for uniquely defining a single tree for all taxa.

**Results:**

We establish theoretical bounds on the impact of missing data on decisiveness. Results are derived for two contexts: a fixed taxon coverage pattern, such as that observed from an already assembled data set, and a randomly generated pattern derived from a process of sampling new data, such as might be observed in an ongoing comparative genomics sequencing project. Lower bounds on how many loci are needed for decisiveness are derived for the former case, and both lower and upper bounds for the latter. When data are not decisive for all trees, we estimate the probability of decisiveness and the chances that a given edge in the tree will be distinguishable. Theoretical results are illustrated using several empirical examples constructed by mining sequence databases, genomic libraries such as ESTs and BACs, and complete genome sequences.

**Conclusion:**

Partial taxon coverage among loci can limit phylogenomic inference by making it impossible to distinguish among multiple alternative trees. However, even though lack of decisiveness is typical of many sparse phylogenomic data sets, it is often still possible to distinguish a large fraction of edges in the tree.

## Background

Ready access to vast sequence databases and new data emerging from large-scale sequencing efforts is transforming efforts to infer the phylogenetic history of species. Efforts to build ever larger subtrees of the "tree of life" are pushing the field to grapple with highly species-rich data sets (plants: 2538 taxa [1]; legumes: 2228 taxa [2]; Asterales: 13,533 taxa [3]; eukaryotes: 73,060 taxa [4]). At the same time, some recent studies exploiting complete genome sequences or genomic libraries include 100s - 1000s of loci [5-11]. The former tend to be data sets with many taxa and few loci; the latter few taxa and many loci. However, the distinction is blurring as larger data sets of both kinds have been constructed using semi-automated assembly pipelines [3,10] and high performance phylogenetic inference packages (e.g. [12]).

Although such vast data sets have effectively solved certain phylogenetic problems, such as the broad outline of yeast phylogeny [5], in other taxa they have uncovered a surprisingly high level of incongruence and uncertainty (reviewed in [13]). Case studies in *Drosophila *[14], rice [1,11,15], house mouse [16], and primates [17] document processes generating incongruence among gene trees in different parts of the genome that contribute nearly as much to the signal as the true species history does. Phylogenetic methods are struggling to keep pace with inferential complications arising from these processes, such as the potential for statistical inconsistency when sequences are concatenated [18,19]. Indeed, a minor paradigm shift appears to be occurring toward retaining the individuality of signals associated with small genomic regions ("gene trees") rather than simply concatenating all sequence data into an overall analysis [20-24]. This approach comprises so-called "supertree" methods [25] in the broadest sense of that term. In phylogenomic contexts this includes methods that explicitly try to build species trees while reconciling gene trees by modeling processes at the interface between phylogenetics and population genetics, such as lineage sorting [24,26,27], recombination [28] and hybridization [29,30].

In addition to biological processes which evidently confound phylogenomic inference are complexities that stem from the origin of the data themselves. One of the hallmarks of typical phylogenomic data sets is partial taxon coverage among loci–by which we mean not merely missing nucleotides within an alignment but loci for which an entire sequence is missing for some taxa (Sanderson et al. [31]; also referred to as "taxon occupancy" by Hejnol et al. [10]). Missing data arise from causes including biases in sequence databases, the technicalities of sequencing strategies, and real evolutionary processes of sequence divergence or gene loss. Whenever sequences from genomic libraries such as ESTs or BAC-ends provide only partial coverage of a genome, the mismatch between regions covered in different species will generate missing data. Whole genome shotgun sequencing using technologies that assemble at low coverage values will likewise entail missing data, especially at broader phylogenetic scales. Even finished whole genomes can fail to have homologs in a collection of species for various reasons ranging from accelerated sequence divergence to gene loss [32]. It is not unusual for finished genomes to have numerous genes with no detectable homologs in any other taxon (e.g., the maize genome has 2000 unique genes not found in close relatives: [33]).

The consequences of missing data for phylogenetic inference were first studied in the context of integrating fossil taxa, in which there are typically large subsets of missing morphological data, with more complete extant taxa [34-37]. More recently the impact of missing data on accuracy of inferred trees has been examined in the more general context of molecular and phylogenomic data sets [11,38-41] where missing data arises for a variety of reasons. Collectively this body of work treats missing data at the scale of individual cells in a taxon by character data matrix or sequence alignment up to entirely missing sequences for some taxa in a supermatrix or supertree setting, which is the phylogenomic context we consider here. Conclusions about the negative impact of missing data on accuracy have ranged from optimistic [37,42,43] to somewhat more cautious [38,41]; it is possibly fair to say that many empirical studies have argued that the impact of missing data tends to be unbiased and can be ameliorated by sufficient overall quantity of data (e.g., [10]), and most simulation work agrees (see exceptions: [35,41]). However, there has been frequent speculation about the distribution of missing data in the matrix (e.g. its "evenness", [38]: page 546; [10]: SOM). General conclusions have remained elusive both because of lack of characterization of this distribution and the difficulty of separating the usual problems of phylogenetic inference–error and bias caused by the data–from the simple *absence of data*.

In the phylogenomic context of multiple loci, a natural question is when can phylogenetic information from different loci be combined to produce a good tree with all taxa present, even when taxa are missing for some loci? Ideally this question should be abstracted away from the details of tree reconstruction methods. We derived some formal results recently for a relatively simple notion of combinability - "decisiveness" ([44]; note, Goloboff [45] used this term with a very different definition). A pattern of taxon coverage is decisive for some tree on all the taxa in a multi-locus data set if that tree is uniquely defined by combining the (correctly inferred and perfectly concordant) trees from the separate loci. Our previous paper [44] focused on a strong sense of decisiveness: decisiveness *for all trees*. In this paper we also consider weaker notions of decisiveness, derive further results on decisiveness in the important context of sampling from genome-scale data, and examine the utility of the concept in real phylogenomic data sets. The theory will permit us to make relatively precise statements about matters such as the amount and distribution of missing entries, at least within bounds. The scale of phylogenomic data sets and the sampling properties of genomic sequencing protocols is likely to increase the relevance of our mathematical theory.

## Methods

### Overview

**Figure 1:**
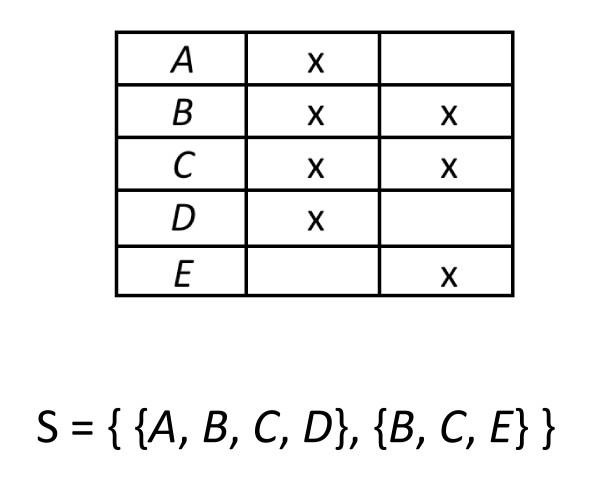
**A simple taxon coverage pattern**. The pattern is shown in both set notation and the more conventional notation as a matrix of taxa by loci. The taxon coverage density is 0.70.

This section outlines several mathematical results, so we begin with an intuitive description of its main point. We assume a phylogenomic data set consists of *k *loci (however defined) and *n *taxa. Loci with data present for fewer than *n *taxa have "missing data". Missing data across the entire data set can thus be characterized by the pattern of "taxon coverage", the set of sets of taxa present for each locus or tree (Figure 1). The basic idea of the paper is that we would like taxon coverage to be "decisive" for the true tree of all *n *taxa. This means that, taken together, all the subtrees of the true tree corresponding to the partial taxon sets at the different loci should be sufficient to uniquely define the true tree. Surprisingly, this is not true for many patterns of partial taxon coverage. This goal is independent of the sequence data, i.e., whether the loci agree on one tree or contain sufficient data to infer any tree. Setting those issues aside lets us isolate the effects of taxon coverage itself. Without sufficient coverage, even the best data will fail to recover a tree even if the underlying data themselves are of impeccable quality.

Questions about taxon coverage arise in at least two contexts. In the first, taxon coverage is given a priori. This might happen when a data matrix has already been assembled from GenBank, or in an examination of a data set from the literature. In this case, the true tree is unknown, and the key question is whether the data set is decisive for all trees, some trees, or no trees. In the second context, taxon coverage can be modeled as the outcome of a sampling process. This might result from a sequencing strategy, for example, that induces a certain quantity of missing data, but for which those data are more or less randomly distributed. Here the pertinent question is how many loci must be sampled to guarantee decisiveness for the unknown true tree.

### Definitions

We begin by recalling some basic definitions from phylogenetic theory. Following Semple and Steel [46], given a set *X* of taxa, a *phylogenetic X-tree *is a (unrooted) tree *T* in which the leaves, or terminal taxa, of *T* consist of the *label set X *(taxon names) and all the remaining vertices (nodes) of *T* are unlabelled and have at least three incident edges. *T *is *binary *(completely bifurcating) if every internal vertex has exactly three incident edges. For a phylogenetic tree *T* and a subset *Y *of *X*, let *T*|*Y *denote the induced phylogenetic tree on leaf set *Y *(the tree obtained from the minimal subtree connecting *Y *by suppressing any vertices of degree two). We will let *n *= |*X*| throughout. A *quartet tree *is a binary phylogenetic tree on four leaves. For such a tree with leaves *a*, *b*, *c*, *d*, we write *ab*|*cd *if the internal edge of the tree separates the pair *a*, *b *from *c*, *d*.

A *rooted phylogenetic tree *is a tree obtained from a phylogenetic tree by deleting some leaf and its incident edge and regarding the other endpoint of that edge as a *root *vertex. Rather than defining the notions that follow for both rooted and unrooted phylogenetic trees separately, we will define them just for unrooted trees; the analogous definitions for rooted trees follow immediately by regarding any rooted tree on a set of *n *taxa as an unrooted phylogenetic tree on *n*+1 taxa, the additional taxon being some fixed reference outgroup (real or hypothetical).

We now define some key concepts. Let *Y *be the (label) set of taxa with nonmissing data for some locus (*Y*_*i *_if locus *i *is specified).

**Figure 2:**
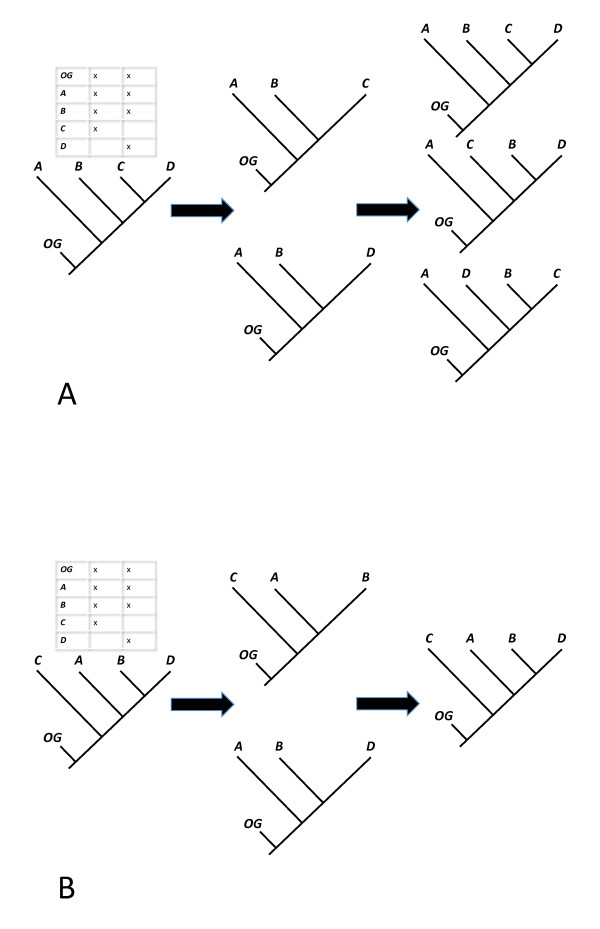
**Taxon coverage and defining trees**. **A**. For the original tree and partial taxon coverage shown on the left, the two induced subtrees (middle) do not define the original tree, because other trees (right, middle and lower) also display these subtrees. **B**. In this case, the middle two subtrees do define the original tree. Notice that the taxon coverage pattern is the same in both cases. This coverage is phylogenetically decisive for the original tree in B, but not in A. Thus, it follows that this taxon coverage is not phylogenetically decisive *for all trees*.

• Whenever *Y *⊆ *X*, we say that a binary phylogenetic *X*-tree *T displays *a binary phylogenetic *Y*-tree, *T*' if *T*|*Y *= *T*'. More generally, for *Y *⊆ *X*, we say that a phylogenetic *X*-tree *T displays *a phylogenetic *Y*-tree, *T*' if *T*|*Y *either equals *T*' or is a resolution of that tree (i.e. all the splits of *T*' are contained in *T*|*Y*).

• We say that a collection of phylogenetic trees *T*_1_,...,*T*_*k *_*defines *a phylogenetic *X*-tree *T *if *X *is the union of the leaf sets of the trees *T*_1_,...,*T*_*k *_and there exists one, and only one phylogenetic *X*-tree that displays these trees, and this tree is *T*. This implies *T *that must be binary.

• An internal edge *e *of a binary tree *T *is said to be *distinguished *by some other tree if both trees display a quartet tree *xy*|*wz *but the tree *T/e *(the tree obtained from *T *by collapsing *e*) does not display this quartet (this means that *x*, *y*, *w*, *z *comes from each of the four subtrees of *T *incident with *e*). There is an analogous notion of 'distinguished' for rooted trees based on rooted triples rather than quartet trees. For example, in Figure 2A the internal edge below the clade C, D of the original tree (left) is not distinguished by either of the two subtrees (middle) shown; however in Figure 2B all three interior edges of the original tree (left) are distinguished by the subtrees (middle).

• For a collection **S **= {*Y*_1_, ..., *Y*_*k*_} of subsets of *X*, we say that **S **is the *pattern of taxon coverage *or just *taxon coverage *(Figure 1). Any taxon found in all *k *subsets is called a *reference taxon*. The taxon coverage *density *is .

• We say that **S ***is decisive for a phylogenetic tree T *provided *T*|*Y*_1_,...,*T*|*Y*_*k *_defines (Figure 2).

• We say that **S **is *phylogenetically decisive for all trees *if it satisfies the following property: If *T *and *T*' are binary phylogenetic *X*-trees, with *T*|*Y *= *T'*|*Y *for all *Y *∈ **S **then *T *= *T*'. In other words, for *any *binary phylogenetic *X*-tree *T*, the collection of induced subtrees {*T*|*Y*:*Y *∈ **S**} defines *T*.

These last two definitions are from [44], although we now adopt the convention of saying "decisive for all trees" instead of merely "decisive". Notice that a pattern of taxon coverage that is decisive for a given tree can involve far fewer loci than any pattern of taxon coverage that is decisive for all trees; for instance, for every binary phylogenetic tree *T*, there is a set **S **of just *n *- 3 quadruples (set of four taxa) for which **S **is decisive for *T *[47] but any set of quadruples that is decisive for all trees must have size that is at least cubic in *n *(this follows from Theorem 1, below).

Notice that there is a subtle but fundamental distinction between questions about whether an *arbitrary *collection of input trees, *T*_1_,...,*T*_*k*_, defines a unique tree, *T*, on the one hand, and whether a *pattern of taxon coverage*, **S **= {*Y*_1_,...,*Y*_*k*_}, together with a tree, *T*, induces a set of subtrees, *T*|*Y*_1_,...,*T*|*Y*_*k*_, that uniquely defines *T*. Intuitively, in the first case the focus is on the subtrees, whereas in the second the focus is the pattern of taxon coverage. The term "define" refers to the first context; "decisive" to the second. What does it mean when taxon coverage is not decisive for all trees? Then there exists two or more distinct trees which cannot be differentiated from one another based only on the subtrees induced by the taxon coverage pattern, because these trees induce the same set of subtrees. Of course, these may or may not include the "true tree" for any given problem in phylogenetic inference, but surely it is risky to be unable to distinguish among possibilities in case the true tree is involved.

### Context 1: Taxon coverage is fixed a priori

**Figure 3:**
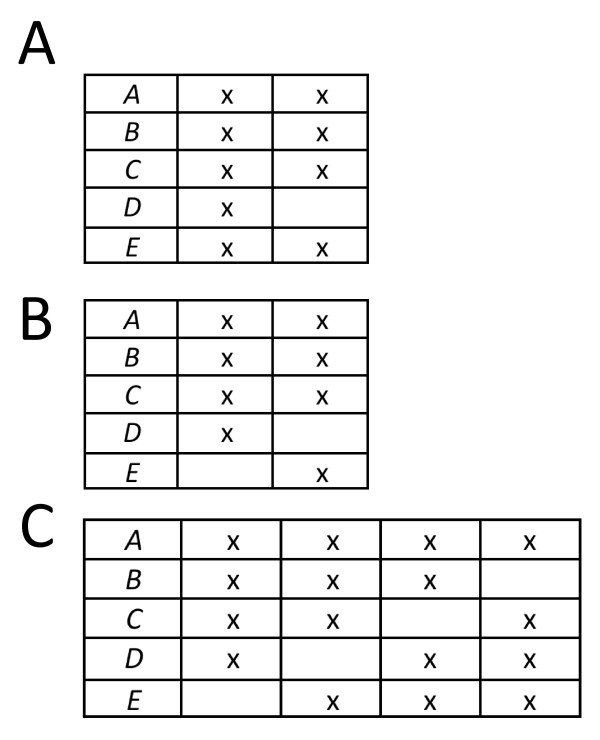
**Taxon coverage patterns and decisiveness**. **A**. Trivially decisive for all trees because one locus contains all taxa and thus by itself satisfies the required four-way partition property. **B**. Not decisive for all trees. For two loci only two quartets are induced by any tree, and although two are *necessary *to define a tree, these two do not suffice. We also know that all triples must be present as a necessary condition for decisiveness for all trees, but the triple {*C, D, E*} is not present in any column. **C**. Decisive for all trees because there is a reference taxon and every quadruple of taxa containing the reference pattern is seen among the loci, which satisfies a sufficient condition.

In this context, we assume **S **is given. Steel and Sanderson [44] established exact conditions for when a pattern of taxon coverage, **S**, is decisive for all (unrooted) trees. Briefly, for every four-element partition of the label set, there must be a quadruple of taxa in some set in **S **such that each element of the quadruple is found in a different element of the partition. In general testing this "four-way partition" condition is computationally expensive, as the number of four-way partitions is exponential in the size of the label set. Easier to compute are (i) a *necessary *condition, which is that for each triple (set of three taxa) there must be at least one taxon set in **S **containing that triple [48], and (ii) a *sufficient *condition, which requires that, assuming a reference taxon exists, every quadruple containing that reference taxon has at least one taxon set in **S **containing this quadruple. See Figure 3 for some simple examples. The necessary condition can be quickly used to determine when taxon coverage is *not *decisive for all trees, and the fraction of triples present may provide an indication of the impact of low taxon coverage. Below we give several empirical examples.

It is also possible to derive some lower bounds on the number of loci required for phylogenetic decisiveness for all trees. We begin with the simpler case of subtrees defining a tree. A necessary condition for an arbitrary collection of subtrees to *define *a tree is given by [49]: if *T*_1_,...,*T*_*k *_define a phylogenetic tree with *n *leaves then:(1)

where *ι*(*T*_*j*_) refers to the number of internal edges of *T*_*j*_. For example, at least *n*-3 quartet trees are needed to define a tree with *n *leaves. A similar necessary condition holds for decisiveness for a given tree. Since *ι*(*T*_*j*_) is at most the number of leaves of *T*_*j *_minus 3 it follows that if **S **= {*Y*_1_,...,*Y*_*k*_} is phylogenetically decisive for *T *then:(2)

[50], where *n*_*j *_= |*Y*_*j *_|. As noted in this last paper, Inequality (2) is not tight in the following sense: certain nine-leaf binary trees cannot be defined by any two six-leaf subtrees, even though Inequality (2) is satisfied since (6-3) + (6-3)≥ (9-3).

A consequence of Inequality (2) is that if the sets *Y*_1_, ..., *Y*_*k *_each have size at most *m *then a lower bound on the number of loci required for decisiveness for a given tree is:(3)

Similar bounds can be obtained for phylogenetic decisiveness for all trees.

**Theorem 1 ***If a collection *S = {*Y*_1_, ..., *Y*_*k*_} *of subsets of X is phylogenetically decisive for all trees then:*.

where *n*_*j *_= |*Y*_*j*_|. In particular, if each of the sets in **S **has size at most *m *then:

(See Appendix for proof). This provides a necessary condition, or lower bound, for phylogenetic decisiveness for all trees. Roughly speaking, if some fraction of taxa, *f *= *m*/*n*, is present for each locus, then we need at least (1/*f*)^3 ^loci for decisiveness for all trees, while at least (1/*f*) loci are required for decisiveness for a given tree.

#### Partial decisiveness

**Figure 4:**
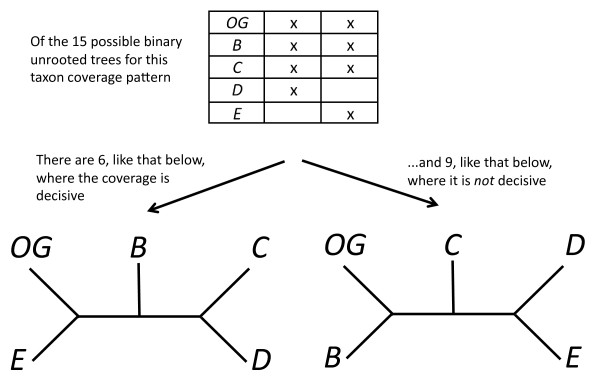
**Partial taxon coverage**. Shown is an example in which taxon coverage is not decisive for all trees, but it is decisive for 6 out of 15 possible rooted trees for 4 taxa. This is illustrated for the equivalent 15 unrooted trees, by taking advantage of the "trick" that there is an outgroup taxon, *OG*, that is common to both trees (a "reference taxon"), and thus we can regard it as the root. Then we can use known results [46] to determine whether the subtrees induced by the taxon coverage define the tree (and thus enumerate for which trees the coverage pattern is decisive).

If we knew *T*, it would be sufficient to use results on defining trees to describe the taxon coverage needed to define *T *from subtrees. In practice we do not, and to play it safe, we require sufficient taxon coverage for all *T *(decisiveness for all trees). This may be too strong a requirement. Perhaps it is sufficient for taxon coverage to be decisive for *most *trees, assuming then that the true tree is likely among this set of trees. Figure 4 shows an example in which taxon coverage is not decisive for all trees (easy to see because not all triples are present), but is decisive for 6 out of 15 possible rooted trees for 4 taxa. This example suggests it would be useful to enumerate or estimate the fraction of trees for which *S *is decisive.

Determining when taxon coverage is decisive for only some trees involves an important open question. When exactly does a set of subtrees define a tree? The bound stated earlier does not specify *how *to tell if some set of *n*-3 or more quartet trees defines a tree. The answer is known for rooted [47], but not for unrooted trees. A collection of rooted trees defines a tree if two conditions are met: each subtree is displayed by the tree, and each internal edge of the tree is distinguished by an internal edge of at least one of the input trees ([47]: see definition above). In contrast to the rooted setting, distinguishing all the edges of an unrooted tree is only a necessary (but not sufficient) condition for a collection of unrooted trees to define that tree. To proceed, we therefore restrict attention to the rooted case, which is equivalent to assuming there exists in the data set a reference taxon which can provisionally be used to root the input trees (in an artificial but useful sense of "rooting").

For rooted trees, the fraction of trees, ∏_*D*_, that are decisive for a given pattern of taxon coverage could be determined by checking these conditions for all possible trees. For small sets of taxa complete enumeration is feasible. For larger data sets, the true fraction of trees for which the taxon coverage is decisive can be estimated from the fraction in a large random sample of trees (under a uniform/equiprobable distribution of trees).

However, the condition of distinguishability of edges suggests a potentially more sensitive measure of "partial" decisiveness: the probability of an edge being distinguished on a tree for a given pattern of taxon coverage, ∏_*d*_. This is the fraction of edges on the tree that are distinguished by subtrees induced by the taxon coverage pattern, averaged across all trees, or a random sample of trees. We examine these measures of partial decisiveness in empirical examples below.

### Context 2: Taxon coverage is the product of a random sampling process

In this context the taxon coverage is not fixed but instead is determined by a random process of sampling. This might be a reasonable model for data assembled from partial genomic coverage, for example. The question we want to address is how many loci must be sampled to guarantee with some high probability that the taxon coverage will be decisive for some unknown tree *T*. In this context we need not require that it be decisive for all trees, because the results we derive will hold for *any *tree *T *and the true tree will be one of these. This is a subtle difference compared to the first context of a fixed taxon coverage pattern, where it made sense to require decisiveness simultaneously for most if not all trees. One way to understand this is to realize that because the sampling scheme is generating random patterns of taxon coverage, there is a chance that they are decisive for any particular true tree, and the probability calculations will be derived on this assumption. [If there were reason to think that two or more trees are "correct", and we want to require the random taxon coverage will define all of them, we might insist on decisiveness for all trees in this context too–see Discussion for possible examples.]

Let *T *be an unknown binary phylogenetic *X*-tree, and suppose we independently sample *k *random subsets *S*_1_,...,*S*_*k *_of *X *according to the following process: For each *i*, taxon *x *is included in *S*_*i *_with probability *p*(*x*), independently of other taxa. We will first consider two cases:

• **Uniform coverage **(***UC***): *p*(*x*) = *p *for all taxa *x *in *X*;

• **Uniform (+1 complete) coverage(*UC***^+1^**): ***p*(*y*) = 1 for one taxon *y *in *X*, and *p*(*x*) = *p *for all other taxa *x *in *X*. This is the case in which a reference taxon is present. Later we will discuss possible extensions of this model to allow variable taxon coverage probabilities across a tree.

Recall that taxon coverage pattern leads to a set of induced subtrees for each locus, *T*|*S*_1_,...,*T*|*S*_*k*_. Previously we assumed there was no conflict among these induced trees–they could be reconstructed accurately, but here we can obtain some results even if these induced trees are not fully resolved. We use a simple random model for lack of resolution in the induced trees. It does not explicitly model the possible sources of this loss of resolution, such as multiple hits in sequence data, but it provides a tractable starting point. Assume for some fixed probability *q *that each edge in *T*|*S*_*i *_(for each *i*) is retained with probability *q *or collapsed with probability 1-*q*, and this is done independently across the edges of *T*|*S*_*i*_, and across the different induced trees. Let  denote the resulting set of partially-resolved induced subtrees of *T*. Let *E*_*k*_(*T*) be the event that  defines *T*.

We are interested in computing bounds on the probability **P**(*E*_*k*_(*T*)) of this event that depend just on *p*, *q *and the total number of taxa *n *=|*X*|. It is useful to distinguish two further cases:

• **Rooted: ***T *(and thereby the induced subtrees) are rooted trees;

• **Unrooted: ***T *(and thereby the induced subtrees) are unrooted trees.

Let *R*(*n*) denote the set of rooted binary trees on *n *leaf taxa, and *U*(*n*) the set of unrooted binary trees on *n *leaf taxa. Note that by rooting a tree on an arbitrary taxon we can view *U*(*n*+1) as equivalent to *R*(*n*). Moreover, in what follows the *UC *condition applied to *R*(*n*) is essentially equivalent to the *UC*^+1 ^condition applied to *U*(*n*+1).

**Theorem 2 **(i). For any rooted tree *T *in *R*(*n*), under *UC *(or an unrooted tree T in *U*(*n*+1) under *UC*^+1^) we have: P(*E*_*k*_(*T*))≥ 1-(*n*-2) · (1-*p*^3^*q*)^*k*^. Consequently, for *ε *> 0, if .

Moreover, for any unrooted tree *T *in *U*(*n*+1) under *UC*, P(*E*_*k*_(*T*)) ≥ 1 - *ε *holds if the term *p*^3 ^in the above inequality is replaced by *p*^4^.

(ii). Conversely, for any *ε *> 0, if  then there exist (exponentially many) choices of *T *∈ *R*(*n*) under UC (and trees in *U*(*n*+1) under *UC *or *UC*^+1^) for which P(*E*_*k*_(*T*)) < 1 - *ε *(See Appendix for proof).

Together these conditions give (respectively) upper and lower bounds on the number of loci needed for decisiveness relative to a single (unknown) tree. The precise number depends on the shape of the tree.

#### Remarks

When *q *= 1 the event *E*_*k*_(*T*) is equivalent to the event that **S **= {*S*_1_,...,*S*_*k*_}is phylogenetically decisive for *T, but for q < 1 the event is a stronger condition*. Also, Theorem 2 readily extends to allow the probabilities of taxon coverage to vary across the tree, albeit at the expense of more complicated formulae. However, there is one simple case where the same formulae apply: Part (i) holds as stated if, in the Uniform Coverage conditions, the equality *p*(*x*) = *p *for all taxa *x *is weakened to *p*(*x*) ≥ *p *for all *x *(similarly, part (ii) holds if we impose an inequality in the reverse direction).

We can also consider models that explicitly reflect how taxon coverage is often much more thorough within a clade than across the entire tree, by considering the following clade-specific condition:

• **Variable coverage (*VC*): ***p*(*x*) ≥ *p *for all taxa *x *in clade *C*, and *p*(*x*) ≥ *p' *for any taxon *x *not in *C *(where *p*' <*p*).

Suppose that we have a rooted tree *T *in *R*(*n*), and for each of its *n-*2 nontrivial clades, we independently generate at least *r *taxon sets, where each taxon is included or not in each taxon set indepedently with a probability *p*(*x*) that satisfies the *VC *condition for that clade. Repeat this independently for all the *n*-2 clades of *T *(thus the resulting total number *k *of taxon sets must be at least *r*(*n*-2)). Then the proof of Theorem 2(i) easily modifies to show that P(*E*_*k*_(*T*)) ≥ 1- *ε *provided that .

## Empirical examples

We explore these theoretical results in several data sets derived from a diversity of phylogenomic data. We assess whether necessary and sufficient conditions for decisiveness for all trees are satisfied using our theoretical results. We also estimate the partial decisiveness quantities ∏_*D *_and ∏_*d *_using simulations, because no analytical results are known. We estimated these quantities for each data set using 1000 replicate simulated random trees generated using PAUP*'s equiprobable option [51]. Since the two measures of partial decisiveness can only be estimated on rooted trees, we estimate these quantities in a "reference subset" of the original data set. A reference subset is found by first identifying the taxon with the most loci present in the taxon coverage pattern, pruning all loci not present in this taxon from the entire matrix, and then removing any taxa that have been rendered data-less by this operation. This generates a data set with a reference taxon in the sense defined earlier, which can then be used to artificially "root" the analysis. Finally, we assess the bounds on data set size implied under random sampling of loci. In those analyses we assume the unrooted case without a reference taxon and require an error probability no greater than 5%.

### Data mining examples

Driskell et al. [6] mined GenBank to build a sparse data set of 251 loci and 69 green plant taxa with a coverage density of 15% (Table 1). A necessary condition for decisiveness for all trees is the presence of all triples: since only 84% are present, the taxon coverage cannot be decisive for all trees. Indeed for the reference subset the coverage is only decisive for 2% of trees, so the probability of decisiveness for the true tree is quite low. However, on average 95% of internal edges across all trees can be distinguished by the taxon coverage. Thus, the coverage is likely inadequate to build the complete binary tree, but there is reason to be optimistic about most of it, at least from the perspective of missing data.

**Table 1:** Taxon coverage and decisiveness parameters for five data sets.

Taxon	Source	Taxa	Loci	Coverage density	Fraction of triples	∏_*D*_	∏_*d*_
Green plants	Driskell et al. 2004	69 (69)	251^a ^(220)	0.15 (0.15)	0.84 (0.84)	0.019	0.95
Pleurodira	PhyLoTA ^b^	44 (37)	15 (12)	0.26 (0.33)	0.37 (0.63)	0.000	0.82
Amorpheae	PhyLoTA ^b^	64 (64)	3 (3)	0.61 (0.61)	0.84 (0.84)	0.017	0.95
Cactaceae	PhyLoTA ^b^	488 (486)	18 (11)	0.14 (0.17)	0.22 (0.23)	0.000	0.61
Metazoa	Hejnol et al., 2009	94 (94)	1487 (1351)	0.18 (0.19)	0.96 (0.95)	0.340	0.99

^a ^Three phylogenetically uninformative loci removed from original data set of 254 loci ^b ^PhyLoTA database [52]

Values in parentheses correspond to the reference subset of taxa. Fraction of triples is the fraction of all possible triples of taxa that are present for at least some locus (column) of the taxon coverage pattern. ∏_*D *_is the probability the taxon coverage is decisive for the reference subset on a random binary tree. ∏_*d *_is the average number of edges distinguishable on a random binary tree (see text for further details).

**Figure 5:**
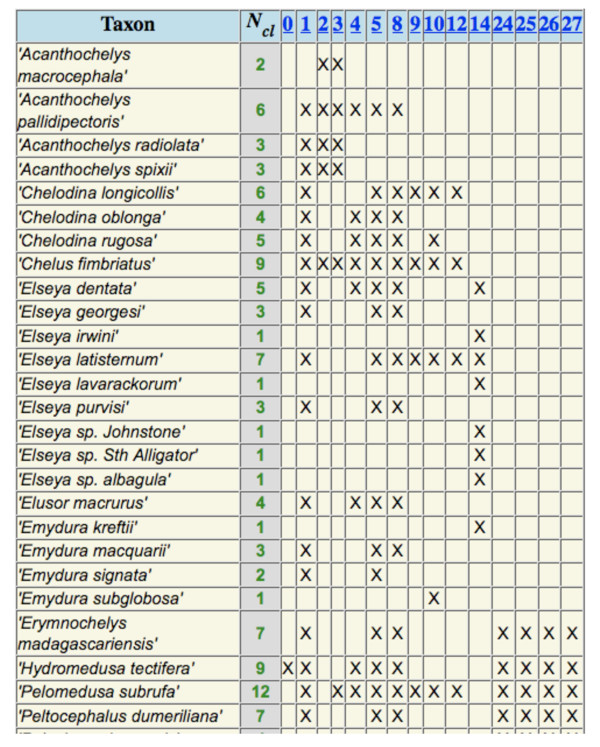
**An empirical taxon coverage pattern**. This example was extracted from the PhyLoTA database (therein called the "data availability matrix"), for sequenced loci for the vertebrate clade Pleurodira (side-necked turtles). Only some of the taxa are shown.

We also examined three data sets extracted from the PhyLoTA database [52], a tool for phylogenomic data mining of GenBank. These were Pleurodira (side-necked turtles: see Figure 5), Cactaceae (the cacti), and Amorpheae (a clade of legumes), all based on data from GenBank release 168 (Table 1). None of the three were phylogenetically decisive for all trees, because none exhibited all possible triples of taxa. The probability of the taxon coverage pattern being decisive for a random tree for their reference subsets was quite small, ranging from 0 to 1.7%. On the positive side, the fraction of edges that can be distinguished on average for a random tree is quite high, ranging from 61% to 95%.

However, the structure of the largest matrix, for Cactaceae is sobering. If we were to continue sampling loci with the same coverage density (14%) in the hopes of constructing a data set with high probability of being decisive for the true tree (Theorem 2, with perfect resolution), 2932 loci would be minimally necessary, and 23,891 would suffice. Of course, these results presuppose something like a uniform distribution of sampling effort across taxa, which is more likely to be found in genomic data sets than database mining studies. Nonetheless, these bounds are suggestive.

Finally, in this category we turn to several studies with very large taxon sets, for which we can only apply some of the simple results in Theorem 1. Goloboff et al. [4] assembled a supermatrix of 13 loci and 70,060 taxa. The locus with largest taxon coverage included ~20,000 taxa. By Theorem 1 the number of loci would have had to exceed 43 for phylogenetic decisiveness for all trees. Since most of the loci were sampled for far fewer than this number of taxa, a more accurate lower bound is probably higher.

This analysis, with coverage density of 8%, may be characteristic of taxon rich phylogenetic analyses published recently in which overall density is low, but a few loci have high coverage densities. For example, in [2] (2228 taxa of Leguminosae) and [3] (4954 taxa of Asterales) the locus with best coverage, nuclear ribosomal ITS, actually covered more than 73% and 84% of the label sets respectively, such that by Theorem 1 the lower bound on the number of loci is small, less than 3. However, in both cases, the ITS sequences were aligned by relatively "heroic" methods, and a more conservative procedure might have kept many separate taxonomically overlapping ITS alignments instead of one large one. We reported results from such an analysis (so-called "sparse" analysis in [2]) that ultimately included 1794 taxa in 72 loci. By Theorem 1 the lower bound for decisiveness for all trees in this case, assuming *n/m *= 1794/286, where 286 is the largest cluster (*trnL*), would have been 247 loci, far more than we had.

### Partial genome sequences from shallow libraries

Hejnol et al. [10] constructed a large, sparse phylogenomic matrix of 94 taxa and 1487 loci derived mainly from ESTs. This is an expanded analysis of the widely cited phylogenomic study of 70 metazoan taxa [8]. Mean taxon coverage density is 18% across all loci, with the maximum density of 83% in one locus and a long tail of very low coverage loci. The given taxon coverage is not decisive for all trees, because it only includes 96% of possible triples (Table 1). *Homo sapiens *is present in 1351 of the loci. If we restrict the data to this reference subset, we can "root" the analysis with *Homo *as a reference taxon and test for a sufficient condition for decisiveness for all trees (a reference taxon plus all triples present: see above). For these loci there are still 94 taxa and 96% of possible triples are present. This fraction *might *have increased to 1 if the number of taxa in the new matrix had been less. This data set stands out by having a relatively high probability of decisiveness for random trees (34%), and a 99% chance of distinguishing a random edge in a tree. Under random taxon coverage (perfect resolution), by Theorem 2 the lower and upper bounds on loci are 1095 and 7148, which spans the actual data set.

### Partial genome sequences from deep genomic libraries

Some genomic libraries are "deep", providing 10s to 100s of thousands of sequences, but still only partially cover a genome. Cranston et al. [11] analyzed data from BAC-end sequences from 10 species of *Oryza *including the complete genome of *O. sativa *and nine deep libraries averaging ~90,000 sequences per species. Collectively, these covered 44% of the CDS (coding regions) in *O. sativa*, but because of the random nature of library construction, of course, the pattern of overlap varied from species to species. The data were further filtered to include only single copy exonic regions with homology to *O. sativa *and having taxon coverage of at least four taxa, a total of 9481 exonic regions.

*A posteriori *the pattern of taxon coverage is trivially decisive for all trees because there are 6 loci (out of 9481) that have complete coverage–which automatically satisfies the four-way condition described earlier. An additional 60 loci are only missing one taxon. However, on average the taxon coverage among these exons averaged 48.3%. By Theorem 2 this value under random sampling and full resolution implies lower and upper bounds of 35 and 91 loci for decisiveness. However, if we consider partial resolution and if only one internal branch is present on average out of the 8 internal branches, the bounds increase to 292 and 742. Among a subset of loci most relevant for 6 of the species (the AA genome group) fully one quarter produced completely unresolved trees (the mean length of aligned exons was only 350 nt), so this is clearly an issue.

### Complete genome sequences

Phylogenomic data sets constructed from complete genome sequences should suffer least from taxon coverage problems. We used Hahn et al.'s [32] analysis of gene families constructed from the 12 *Drosophila *complete genome sequences then available. Data were downloaded from Hahn's FRB Database http://www.indiana.edu/~hahnlab/fly/DfamDB/drosophila_frb.html and parsed to quantify taxon coverage. Loci for which there was at most a single copy of a gene present were retained. Of these 13,677 loci, 6979 had some taxa missing. The average taxon coverage was 66.3%. Note that this number is somewhat inflated because all loci that had only one taxon sampled (singletons) were excluded (Hahn et al. suggested many of these represent annotation errors). Clearly with 6698 loci having complete taxon coverage, there is no problem with phylogenetic decisiveness for all trees. Moreover, at this rate of taxon coverage on average, by Theorem 2, the number of randomly sampled loci needed for decisiveness for the true tree is bounded by 12 - 25 loci for 12 taxa, and even if resolution among trees were dismal (say one branch only out of 9 internal branches) we would have bounds of 127 and 246 loci. Thus if one were setting out to build a phylogenomic matrix with these data, this simple analysis suggests there are plenty of loci available. Interestingly the fractional taxon coverage is so high that even were one able to do this across 1000 species of *Drosophila*, the number of loci is still bounded by 26 - 47.

## Results and Discussion

### Impact of missing data on multi-locus phylogenetic inference

Assessing the effect of missing data on phylogenetic inference has received substantial attention from phylogeneticists over many years (reviewed in [53]), but unambiguous conclusions have been elusive. For some amounts and patterns of missing data, simulation and empirical studies support the conclusion that its impact can be reduced to tolerable levels [37,38]; for other cases, significant problems remain [39-41]. Properties of the data themselves, such as rates of evolution, stationarity, etc., can have a strong impact on the accuracy of inference, irrespective of the amount or pattern of missing data, so attempts to understand the problem at a comprehensive level taking all impacts into account have had poor predictive success.

Our results relate the taxon coverage–the amount and pattern of missing data–to the ability of data to define an unambiguous phylogenetic tree for all the taxa in the input, by separating the impact of missing data from tree reconstruction errors per se. To simplify the problem, we have defined a notion of phylogenetic decisiveness [44], which is separate from any notion of correctness of the individual gene trees based on individual loci.

We infer limits (bounds) on decisiveness. In both the context of a fixed pattern of taxon coverage and a random pattern generated by sampling, there are lower bounds to how many loci must be included for a given fraction of missing data. For the sampling case it is also possible to identify upper bounds so that it is clear when sufficient data are present. For a fixed (i.e., observed) taxon coverage pattern, it is possible to characterize whether it is decisive for some or all possible trees, but computing the latter is nontrivial.

Our results may help clarify why some arguments in the literature appear at odds with each other. On the one hand, Wiens [37] has suggested that it is not the fraction of missing data that is most important, but the overall amount of character data present. The most direct support from this in our results is seen in the random sampling context. According to Theorem 2 (i), for any fraction of missing data (1 - *p*), there will be a number of loci that is sufficient to guarantee decisiveness. Another suggestive bit of evidence is seen in Table 1: Cactaceae and Metazoa have about the same fraction of missing data overall, but because Metazoa have so many more loci, the fraction of triples seen in that coverage pattern is much closer to the minimum level necessary for decisiveness (96% vs. 22%). A caveat is that the number of loci required can be *quite *large if the fraction of missing data is large or the number of taxa large (when *p *<< 1 and or *q *<< 1, the lower bound on number of loci *k *scales as 1/(*p*^3^*q*) (expanding the expression  Theorem 2 part (ii) in a Taylor series approximation)).

On the other hand, Hartmann et al. [39] and Lemmon et al. [41] discuss cases in which missing data are more problematic. Both studies simulated missing data at the sequence level occurring in blocks, which in our context we can think of as "loci". Both attributed errors in phylogenetic inference in part to these patterns of taxon coverage, often in combination with other factors. Our results on phylogenetic decisiveness for a fixed pattern of taxon coverage make it clear that some patterns of taxon coverage can be pathologically bad. Once we cannot count on random taxon coverage to ultimately sample all 4-way partitions, it is easy to imagine patterns in which certain important 4-way partitions are never seen and thus ambiguity remains a real possibility even as the number of loci gets arbitrarily large. The obvious case is just to suppose that we sample many loci but all of them either have {*A, B, C, D*} or {*A, B, C, F*} taxon coverage. This will never be decisive according to the 4-way partition condition. Some of Hartmann et al.'s [39] EST-like patterns of missing data are reminiscent of this case because of blocks of missing data at the ends in many alignments.

### Implications in the context of gene tree discordance

Evidence is mounting that high levels of gene tree discordance is driven in part by real biological processes of lineage sorting, hybridization and recombination [14,16,17,28]. Our analyses have by design set aside the latter issue, and by necessity the former, but incongruence is obviously real. Intuitively, since the lower bounds on the number of loci needed to infer a single tree decisively under random taxon coverage were derived assuming just one tree (see Appendix), we expect it would require many more loci to simultaneously be able to recover more than one tree. Interestingly in the Cranston et al. [11] analysis of rice phylogenomics, 118 out of 236 possible rooted 5-taxon trees were found among the collection of gene trees (some undoubtedly due to error rather than lineage sorting). If lineage sorting is this rampant in a data set, we would need to know bounds not on decisiveness for one tree under random sampling but decisiveness for all trees. In an a posteriori analysis of a given pattern of coverage (as with rice), we can check decisiveness for all trees after the fact, and gene tree discordance provides a strong motivation for requiring this property.

### Implications for next-gen sequencing strategies

Next generation sequence technologies are currently characterized by assembly of a large number of small reads. In phylogenomic analyses in which de novo assembly (without a reference genome) with limited coverage may be unavoidable, partial taxon coverage in the final data set may be the rule rather than the exception [54]. For example, the 1KP project [55] aims to use a short-read high-throughput approach to sequence transcriptomes of 1000 green plant species across 1 billion years of evolutionary history. A combination of partial transcriptome coverage in the final assemblies and lack of homology across such deep divergences will certainly limit the overall taxon coverage densities. Given estimates of this fraction derived from pilot studies it should be possible to use Theorem 2 to predict how much of the transcriptome is needed to anticipate a decisive analysis.

### Does conflict hurt or help?

A particular consequence of Inequality (1) is that a binary phylogenetic tree *T *for a large number of species cannot be defined by a small number of poorly resolved trees. For example, if *T *has 1000 leaves, then we require a total of 997 or more internal edges amongst any set of trees that defines *T*. Now suppose we use a supertree method like MRP (Matrix Representation with Parsimony, see review in [25]) on these input trees. An interesting question is whether Inequality (1) might still hold if we replace the condition that the trees "*T*_1_,...,*T*_*k *_define *T" *by "*T*_1_,...,*T*_*k *_have a unique MRP tree *T"*.

This seems plausible since the two notions are equivalent when the input trees *T*_1_,...,*T*_*k *_are compatible, and it might be supposed that conflict-free data is in some sense 'optimal'. However, it turns out that such a modification to Inequality (1) fails. For example, there is an incompatible set of six trees for 12 taxa, each with just one internal edge, that has a unique MRP tree *T *on those 12 taxa (results not shown); by contrast if we wished to additionally require the input trees to be compatible, we would require at least nine trees (each having one internal edge), not six. In other words, allowing the trees to conflict can in some cases actually 'help' reduce the amount of data required to determine a unique supertree by a method such as MRP.

### Relation to phylogenetic "groves"

**Figure 6:**
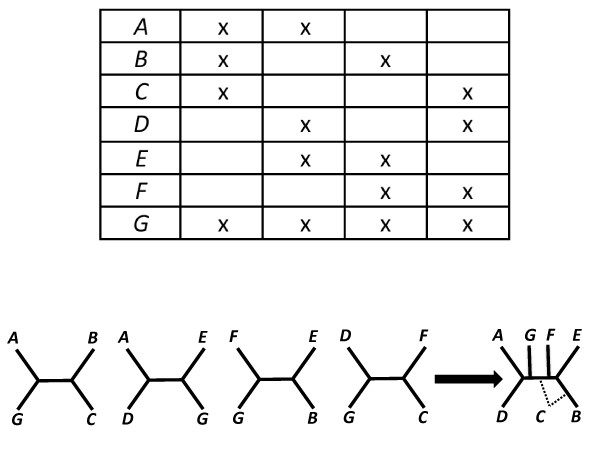
**Decisiveness and phylogenetic groves**. Pattern of taxon coverage shown is not decisive for any tree but is a phylogenetic grove sensu Ané et al. [56]. There are five binary trees like the tree in the lower right, which differ only in the placement of taxon *C *(two of these are indicated by alternative placements of dashed lines). Although taxon coverage is not decisive for any of the five trees, there is no ambiguity about the relationships of the five trees if taxon *C *is ignored. Example modified from [56,57].

Decisiveness for all trees is a strong condition. It is sometimes possible to glean information about a tree that includes all taxa even when the taxon coverage pattern is not decisive for *any *such tree. A weaker sense of decisiveness is embodied in the notion of a "grove" of phylogenetic trees ([56]; reviewed in a less mathematical exposition in [57]). Figure 6 illustrates a taxon coverage pattern that is not decisive for *any *tree. However, for *some *tree(s) it exhibits the property that the collection of trees that display the subtrees induced by the taxon coverage pattern agree on *parts *of the tree. In the example it is only the position of one taxon that is variable among the trees consistent with the induced subtrees. The induced subtrees in this case are said to form a grove, because the trees discovered have information in them that is not contained in any of the induced subtrees alone.

### Future work

Several problems are suggested by this work and its application to empirical studies. First, many real taxon coverage patterns are sparse enough that they are not decisive for all trees. It would be useful to know what data should be obtained to convert an indecisive to a decisive data set, or to convert one that is decisive with low probability to one that is decisive with high probability. In both cases there are natural optimality problems that could be stated that would try to minimize the cost in additional sequencing. Second, along the lines of phylogenetic groves, when a taxon coverage pattern is not decisive for the entire label set, it might well be decisive for a large subset of it. What is the size of the largest label set for which a given pattern of taxon coverage is decisive? In some sense this conveys the "effective tree size" which a taxon coverage pattern can address.

## Conclusions

We have characterized the impact of partial taxon coverage in multilocus phylogenomic data sets in the context of both fixed and random patterns of coverage. For fixed coverage patterns we have determined lower bounds on the number of loci needed for a coverage pattern to be phylogenetically decisive for all trees–that is to allow reconstruction of a unique tree on all taxa irrespective of what the tree is. Most real data sets having reasonably large numbers of taxa are not decisive for all trees, but in these cases we developed methods for assessing the fraction of trees for which they are decisive and the fraction of edges in those trees that can be distinguished. In general, the first measure is still low, but the second can be quite high. For random taxon coverage patterns, such as might be seen in data sets derived from EST or BAC libraries, upper and lower bounds on the number of loci needed for decisive tree inference were obtained. Some real data sets required relatively few loci for decisiveness, particularly those with few taxa and modest levels of missing data, whereas other data sets required tens of thousands of loci when the number of taxa and fraction of missing data was high. Clearly, the pattern of taxon coverage is a factor in large scale phylogenetic inference, regardless of the details of the sequence data themselves or tree reconstruction method.

## Authors' contributions

MJS, MMM, and MS conceived the initial problems, which were then formalized as theorems and proofs by MS. MJS and MMM designed and analyzed the empirical studies and formulated implementations of partial decisiveness measures. MMM wrote code to support the latter. MJS and MS wrote the manuscript, with editing by MMM. All authors read and approved the final manuscript.

## Appendix: Proofs

*Proof of Theorem 1*. If *S *is phylogenetically decisive for all trees then:

In other words, all three-taxon subsets of must be present as a subset of some element *Y *of **S **(this is Lemma 6.2.1 of [48]). Thus:

from which the results stated now follow.

*Proof of Theorem 2*: Part (i). First suppose that *T *is in *R*(*n*). For each internal edge *e *of *T *let *A*_*e *_be the event that *e *is distinguished by at least one of the trees  (for the definition of 'distinguished' see [46]). Let *B*_*e*,*i*_be the event that edge *e *is distinguished by *T*|*S*_*i*_. By Theorem 3 of [47]*T*|*S*_1_,...,*T*|*S*_*k *_defines *T *precisely if each internal edge of *T *is distinguished by at least one of these induced trees. Consequently:(4)

where *E*_*int*_(*T*) is the set of internal edges of *T*, and(5)

By the Bonferroni inequality:(6)

where overline denotes the complementary event. Now, by (5), we have:  and for fixed *e*, the events  are independent events of equal probability; consequently:(7)

Now, if *n*_1_, *n*_2_, *n*_3 _denotes the number of leaves (in *X*) in the three subtrees of *T *that are incident with *e *but which do not contain the root of *T *then:

where the inequality arises because *n*_1_, *n*_2_, *n*_3 _≥ 1. Applying this inequality to (7) we obtain:  and so, by (6):

since a tree in *R*(*n*) has precisely *n *- 2 internal edges, as required. The resulting bound involving *k *now follows by routine algebra.

Turning to the case of an unrooted tree *T *∈ *U*(*n *+ 1), if condition *UC*^+1 ^holds then we can directly apply the result for rooted trees under *UC*. However if the weaker condition *UC *holds for *T *∈ *U*(*n *+ 1)then we use the result (from [47]) that *T *is defined by any set of quartet trees that distinguishes every edge of *T *provided that each quartet contains a fixed leaf. We can then repeat the above argument but at the penalty of increasing the exponent on *p *from 3 to 4.

*Proof of Part (ii)*: Let m = ⌊n/3⌋, and let *T*_1 _be any tree in *R*(*m*). Identify each leaf of *T*_1 _with the root of a separate 3-leaf binary tree to obtain a tree in *T*_2 _in *R*(3*m*). Consider the set *E'* of the *m *newly-created internal edges of this tree. If *n *> 3*m *then form an arbitrary rooted tree from the remaining *n *- 3*m *leaves, adjoin its root, and the root of *T*_2 _to a new root to obtain a tree *T *∈ *R*(*n*) (if *n *= 3*m*, we simply take *T *= *T*_1_). Now each edge *e *∈ *E*' has just one leaf in the three subtrees that are incident with the edge and that do not contain the root of *T*. Thus, for each of these *m* internal edges we have:

and so, by (5), and the independence of *B*_*e*, *j *_for different *j*, we have:

Now, although for a general tree the events *A*_*e *_are not independent, for this particular tree, and for this set *E*' of *m *internal edges, these events are independent. Consequently, by (4),

from which part (ii) follows upon applying the inequality:  This completes the proof.
